# Interleukin (IL)-21 Promotes the Differentiation of IgA-Producing Plasma Cells in Porcine Peyer's Patches via the JAK-STAT Signaling Pathway

**DOI:** 10.3389/fimmu.2020.01303

**Published:** 2020-06-23

**Authors:** Guo Liu, Bin Wang, Qingbo Chen, Yang Li, Baoyu Li, Ning Yang, Shanshan Yang, Shuxian Geng, Guangliang Liu

**Affiliations:** State Key Laboratory of Veterinary Etiological Biology, Lanzhou Veterinary Research Institute, Chinese Academy of Agricultural Sciences, Lanzhou, China

**Keywords:** IL-21, IgA class switch recombination, B cell differentiation, JAK-STAT, porcine Peyer's patches

## Abstract

Secretory IgA is critical to prevent the invasion of pathogens via mucosa. However, the key factors and the mechanisms of IgA generation in the porcine gut are not well-understood. In this study, a panel of factors, including BAFF, APRIL, CD40L, TGF-β1, IL-6, IL-10, IL-17A, and IL-21, were employed to stimulate IgM^+^ B lymphocytes from porcine ileum Peyer's patches. The results showed that IL-21 significantly upregulated IgA production of B cells and facilitated cell proliferation and differentiation of antibody-secreting cells. In addition, three transcripts in porcine IgA class switch recombination (CSR), germ-line transcript α, post-switch transcript α, and circle transcript α, were first amplified by (nest-)PCR and sequenced. All these key indicators of IgA CSR were upregulated by IL-21 treatment. Furthermore, we found that IL-21 predominantly activated JAK1, STAT1, and STAT3 proteins and confirmed that the JAK-STAT signaling pathway was involved in porcine IgA CSR. Thus, IL-21 plays an important role in the proliferation and differentiation of IgA-secreting cells in porcine Peyer's patches through the JAK-STAT signaling pathway. These findings provide insights into the mucosal vaccine design by regulation of IL-21 for the prevention and control of enteric pathogens in the pig industry.

## Introduction

Secretory immunoglobulin A (sIgA) dominates in the mucosal surface, playing a vital role in protecting the mucosal epithelium from toxins and pathogens and maintaining homeostasis at mucosal surfaces (1). In gut-associated lymphoid tissues, Peyer's patches (PPs) are considered as the most important IgA-inducing sites to defend the invasion of enteric pathogens (2). Porcine ileum Peyer's patch (IPP) covers a large area in the small intestine, extending as long as 3 meters. It develops in gestation and even evolves under germ-free conditions (3). The initial role of IPP is to rapidly provide neonatal piglets with large quantities of T cell-independent, undiversified natural sIgA to the gut lumen and a primary IgA response in serum upon colonization (4). Oral administration with toxins, bacteria or viruses can effectively induce immune responses in young pig's PPs, characterized by dendritic cell (DC) and T-cell activation, upregulated expression levels of cytokines and sIgA antibody (5–8), indicating that IPP may play a vital role in intestinal mucosal immune responses against pathogens in pigs. However, the mechanisms in regulating IgA production in the IPP of pigs have not been described in detail.

The generation of IgA antibody occurs through class switch recombination (CSR) in B cells, a form of deletional DNA recombination occurring between switch regions. Three non-coding transcripts produced in this process, germ-line transcript α (GLTα), post-switch transcript α (PSTα), and circle transcript α (CTα), especially CTα, serve as the hallmarks of active IgA CSR *in vitro* and *in vivo* (9). Studies from a mouse model and humans show that IgA is generated through T cell-independent (TI) and T cell-dependent (TD) pathways. Many cytokines and factors, such as B-cell-activating factor (BAFF), proliferation-inducing ligand (APRIL), TGF-β1, IL-2, IL-4, IL-5, IL-6, IL-10, IL-13, IL-15, IL-21, and IFN-γ, benefit the differentiation of IgA^+^ B cells (10). However, the porcine immune system differs greatly from that of mice and humans in terms of Peyer's patches. PPs in humans and mice are discrete follicles, such as those in porcine jejunum; in contrast, porcine IPPs are continuous ones. There are more B cells, fewer T cells, and less lymphocyte trafficking in the IPP (11). Therefore, studies on IgA production in porcine IPP are needed to understand the difference in the mucosal immune response.

In this study, we isolated IgM^+^ B cells from the IPPs of pigs and investigated the key factors and cytokines promoting IgA production. The detailed mechanism responsible for the regulation of IgA was also studied from the aspects of cell proliferation, differentiation and signaling pathways involved. PCR methods to identify the key transcripts, porcine GLTα, PSTα, and CTα, were established for the first time to evaluate porcine IgA CSR. These studies revealed the mechanism of the gut IgA response in pigs, which will ultimately contribute to the mucosal vaccine design in veterinary research.

## Materials and Methods

### Preparation of B Cells

Peyer's patches were collected from the ileum of 4- to 6-month-old healthy Large White pigs. The mucus, intestinal villus, and serosal surface were removed before the separation of lymphocytes. Mesenteric lymph nodes (MLNs) were also collected. These tissues were washed with cold sterile PBS, minced into small pieces and homogenized in a cell separator GentleMax (Miltenyi Biotec). The cell suspension was gently mixed with PBS and passed through a 100 μm cell strainer to exclude the tissue debris. Cells were collected by centrifugation and resuspended in 40% Percoll plus medium (GE healthcare) and laid on the top of 67.5% Percoll plus medium. The lymphocytes were harvested from the interface between the two Percoll layers after centrifugation at 1,800 rpm for 30 min at room temperature. After being passed through a 40 μm cell strainer, the lymphocyte suspension was stained with porcine IgM monoclonal antibody (Washington State University Monoclonal Antibody Center) and then incubated with anti-mouse IgG Microbeads (Miltenyi Biotech). The IgM^+^ B cells were collected through a magnet-based column following the manufacturer's manual.

All animal experiments were conducted according to the Guide for the Care and Use of Laboratory Animals of Lanzhou Veterinary Research Institute, Chinese Academy of Agricultural Sciences, China.

### B Cell Culturing and *in vitro* Activation

Purified IgM^+^ B cells from 2 to 3 pigs were pooled and cultured in RPMI 1640 (Sigma-Aldrich) supplemented with 10% FBS (Gemcell), 10 mM HEPES (Invitrogen Life Technologies), 0.1 mM non-essential amino acid solutions (Sigma-Aldrich), 2% penicillin-streptomycin (Gibco), 100 mg/ml gentamicin (Solarbio) and 50 μM 2-ME (Sigma Aldrich). Swine BAFF (100 ng/mL) and APRIL (100 ng/mL) were used as T cell-independent stimuli. Porcine TGF-β1 (10 ng/mL), IL-6 (50 ng/mL), IL-10 (200 ng/mL), IL-17A (50 ng/mL) and IL-21 (50 ng/mL) were added to the culture medium together with CD40L (1 μg/mL) to evaluate their effects on IgM and TD IgA production in B cells. The recombinant porcine TGF-β1 was purchased from R&D system, while all other factors and cytokines were from Kingfisher Biotech. The JAK-STAT signaling pathway inhibitors solcitinib and fludarabine (Selleck.cn) were added at different concentrations to the culture medium at 24 h before CD40L and IL-21 treatment to test their inhibitory effect.

### Flow Cytometry

To test cell proliferation, IgM^+^ B cells were labeled with CFSE (BD Bioscience) and cultured with CD40L combined with IL-10 or IL-21 for 3 days. Apoptotic B cells were detected by flow cytometry using a FITC Annexin V Apoptosis Detection Kit I (BD Bioscience) according to the manufacturer's instructions. Live cells were determined by size and granularity, measured by forwarding angle scatter (FSC)/90° light angle scatter (SSC) dual parameters, as described previously (12, 13). All flow cytometry analyses were performed on a BD Accuri C6 flow cytometer and analyzed using FlowJo (Version X) software.

### ELISA

After a 5-day culture of B cells with different factors and cytokines, as described before, the expression levels of IgM and IgA in culture medium were measured using a Pig IgM/IgA ELISA Kit (Bethyl Laboratories) according to the manufacturer's instructions.

### Semi-Quantitative PCR and Real-Time Quantitative PCR

To evaluate IgA CSR in IgM^+^ B cells, primer sets were designed based on IgH constant regions of *Sus scrofa* gene sequences (GenBank Accession Number: AB513625 and AB699687). Conventional PCR methods were developed to detect porcine GLTα and PSTα, and a nested PCR method was developed for the detection of swine CTα. Total RNAs were extracted from IgM^+^ B cells after culturing for 3-days using an RNeasy Plus Mini Kit (Qiagen). RNA (200 ng) was reverse transcribed with oligo(dT)_18_ primers. Then, 2 μL of cDNA was applied to conventional PCR (GLTα, PSTα) or nested PCR (CTα) to detect the relative expression levels using specific primers. The PCR amplicons were visualized by electrophoresis on 1.5% agarose gels stained with ethidium bromide and semi-quantified based on their gray density compared to *GAPDH*.

To evaluate the relative expression levels of *PRDM1* (*BLIMP1*), *IRF4, BCL6*, and *PAX5*, single-stranded cDNA was synthesized with a First Strand cDNA Synthesis Kit (Thermos Fisher Scientific). Quantitative PCR was performed using SYBR^TM^ Green Master Mix (Thermo Fisher Scientific) and normalized to *GAPDH* RNA expression. All the primers were synthesized by Genewiz (Jiangsu, China). Detailed information of the primers used for semi-quantitative PCR and real-time PCR is listed in Table 1.

**Table 1:** The sequence of the primers used for PCR in this study.

**Target gene**	**Forward primer (5^**′**^−3^**′**^)**	**Reverse primer (5^**′**^−3^**′**^)**	**Product size (bp)**	**References**
*PRDM1*	ATGACACACAAATCCAGAGCCA	GGGAGTCCAATTTCAGGATTTC	117	This study
*IRF4*	CCGGCCTGTGAAAATGGTTG	GGACGTGGTCAGCTCTTTCA	186	(14)
*BCL6*	GTATCCAGTTCACCCGCCAT	AGGACCGTCTTATGGGCTCT	127	This study
*PAX5*	TGTTTGCCTGGGAGATCAGG	CCGTGGACACTATGCTGTGA	159	This study
*GAPDH*	ACATGGCCTCCAAGGAGTAAGA	GATCGAGTTGGGGCTGTGACT	106	(15)
GLTα	ACTCCAGCTCCTATGCAGCG	ACTAGGGCTCCAGGTTACTGT	242	This study
PSTα	GCACGATTTTCAGTTGGCCC	GCTCTGACGGGAAGAAGTCC	251, 515	This study
CTα	CTGAGGCCGCACCACCAG	AGATGGACACGGACTTGGTG	359	This study
	ACTCCAGCTCCTATGCAGCG	GGGGATGCTCACAGAGGGTA		This study

### Western Blotting

Cultured IgM^+^ B cells were collected and lysed with RIPA lysis buffer (Beyotime Biotechnology). Total cell lysates were mixed with loading buffer, boiled for 10 min, and then subjected to SDS-PAGE electrophoresis. Proteins were transferred onto PVDF membranes (GE Healthcare). After blocking with 5% skim milk, PVDF membranes were incubated with different primary antibodies overnight at 4°C, followed by incubation with peroxidase-conjugated goat anti-Rabbit IgG at room temperature for 1 h. Bands were visualized using chemiluminescence detection reagents (Advansta). All antibodies used in this study were purchased from Cell Signaling Technology, except for the p-STAT3(S727) monoclonal antibody, which was from Abcam.

### Statistical Analysis

Data are shown as the mean ± SD and were analyzed using GraphPad Prism (version 7.0) software (GraphPad). Unpaired *t*-test was used to analyze statistical significance (^*^ indicates *p* < 0.05, ^**^ indicates *p* < 0.01, ^***^ indicates *p* < 0.001, and ^****^ indicates *p* < 0.0001).

## Results

### IL-21 Enhances IgA Production in Porcine IgM^+^ B Lymphocytes

**Figure 1:**
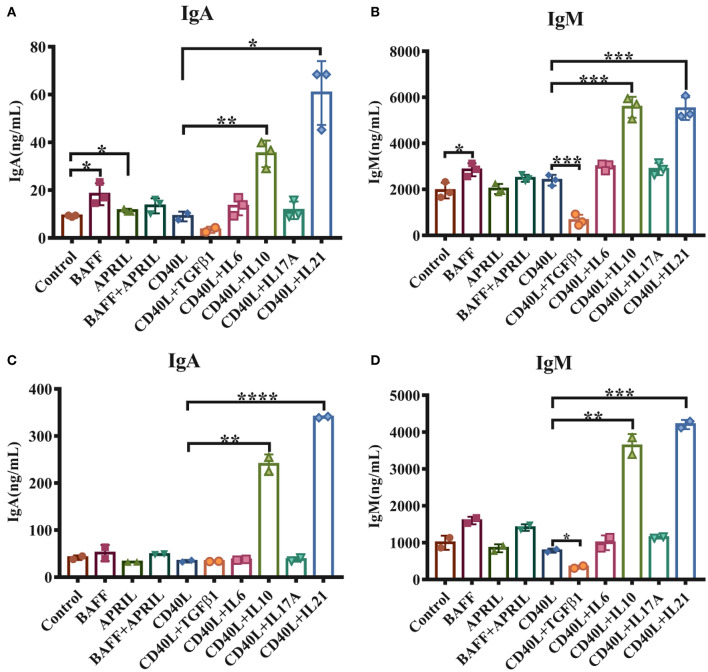
IL-21 increases IgA secretion in IgM^+^ B cells from porcine PPs and MLNs. IgM^+^ B lymphocytes from porcine Peyer's patches and MLNs were isolated and cultured with the indicated factors and cytokines for 5–6 days. The IgA and IgM levels secreted by porcine IgM^+^ B cells from Peyer's patches **(A,B)** and MLNs **(C,D)** were measured by ELISA. Experiments were performed independently in duplicate and triplicate. *p ≤ 0.05; **p ≤ 0.01; ***p ≤ 0.001; ****p ≤ 0.0001.

To investigate the functions of cytokines on porcine intestinal mucosal immunity, IgM^+^ B lymphocytes were magnetically isolated from porcine PPs and incubated with either TI or TD activation factors. The results demonstrated that 20–40% of lymphocytes in PPs were IgM^+^ B cells, and the purity of isolated IgM^+^ B cells reached higher than 90% (Supplementary Figure 1). As shown in Figure 1A, the TI factors BAFF and APRIL slightly promoted IgA production. Regarding TD factors, CD40L alone showed no effect on IgM or IgA production, while IL-21 increased the production of IgA 6-fold and IL-10 upregulated IgA expression levels 3- to 4-fold compared with the CD40L treatment group (Figure 1A). IL-10 or IL-21 enhanced the expression level of IgM 3-fold (Figure 1B). TGF-β1 significantly reduced the IgM expression level but did not affect IgA production.

The impacts of these cytokines on MLN IgM^+^ B lymphocytes were also evaluated, and similar expression patterns of IgA and IgM were observed for stimulation with IL-10 and IL-21 (Figures 1C,D). These data suggested that both IL-10 and IL-21, especially IL-21, played important roles in IgA production in porcine PPs and MLNs.

### IL-21 Promotes the Proliferation and Survival of IgM^+^ B Cells

**Figure 2:**
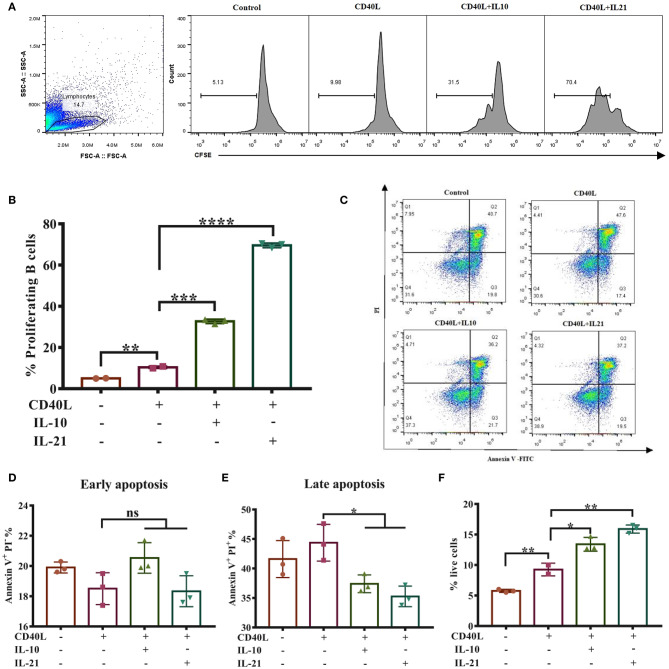
IL-21 promotes IgM^+^ B cell proliferation and inhibits their apoptosis. IgM^+^ B cells labeled with CFSE were cultured alone or with CD40L/IL-10/IL-21 for 3 days, after which the cells were collected and detected through flow cytometry, and the live cell percentage and proliferation rate were analyzed. The representative of three independent experiments was shown for the proliferating cells in each group **(A)**, with quantification of proliferating B cells **(B)**. After culturing for 2 days alone or with CD40L/IL-10/IL-21, IgM^+^ B cells were collected and labeled with Annexin V-FITC and PI and detected by flow cytometry. Representative flow cytometry plots showing apoptotic cells in each group **(C)**, showing quantification of early apoptotic cells **(D)** or late apoptotic cells **(E)**. The percentages of live cells were calculated in each group **(F)**. *p ≤ 0.05; **p ≤ 0.01; ***p ≤ 0.001; ****p ≤ 0.0001.

Considering that cell viability may influence the antibody secretion of B lymphocytes, we next investigated whether IL-10 and IL-21 affect B cell proliferation and apoptosis. The CSFE staining and culture analysis indicated that IL-10 and IL-21 promoted 30 and 70% of total B cell proliferation, respectively, showing a significant increase compared to the treatment with CD40L alone (Figures 2A,B).

B cell apoptosis was analyzed after stimulating cells for 2-days by flow cytometry (Figure 2C). The results showed that IL-10 and IL-21 had no influence on early apoptosis (Annexin V^+^ PI^−^, Figure 2D) but reduced the percentages of late apoptosis (Annexin V^+^ PI^+^, Figure 2E). The percentages of live B cells were improved by CD40L and further increased by the addition of IL-10 and IL-21 (Figure 2F). All these pieces of evidence suggested that both IL-10 and IL-21, particularly IL-21, promoted B cell proliferation and inhibited apoptosis, thus improving IgM^+^ B cell viability.

### IL-21 Induces IgA Class Switch Recombination in Porcine IgM^+^ B Cells

**Figure 3:**
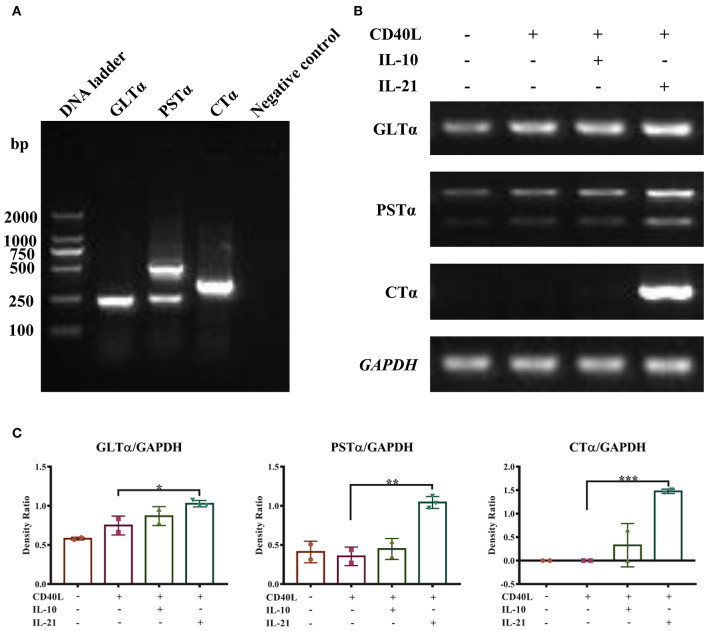
IL-21 induces IgA CSR in IgM^+^ B cells. Semi-quantitative RT-PCR methods were developed to detect the molecular markers for IgA CSR, and representative PCR products of GLTα, PSTα, and CTα are shown **(A)**. IgM^+^ B cells were cultured alone or together with CD40L/IL-10/IL-21 for 72 h, and RNA was extracted. Semi-quantitative RT-PCR was performed to analyze the expression of molecular markers for IgA CSR. Representative results of the expression of GLTα, PSTα, CTα, and *GAPDH* **(B)**, and their density ratios normalized by *GAPDH* **(C)**. *p ≤ 0.05; **p ≤ 0.01; ***p ≤ 0.001.

PCR methods were developed to detect porcine GLTα, PSTα, and CTα. The products are shown in Figure 3A and were verified by DNA sequencing. The detailed DNA sequences of GLTα, PSTα, and CTα were deposited into NCBI GenBank and are publicly available via accession numbers MN629331 (GLTα), MN629332 and MN629333 (PSTα), and MN629334 (CTα). The sequence structures of each transcript are shown in Supplementary Figure 2.

With these (nest-)PCR methods, the IgA CSR influenced by IL-10 and IL-21 was evaluated and normalized to *GAPDH*. The results demonstrated that IL-21 significantly increased the expression of GLTα, PSTα, and CTα (Figures 3B,C), indicating that IL-21 promoted IgA CSR in PP IgM^+^ B cells. To exclude the influence of residual IgA^+^ B cells in IgM^+^ cells from porcine PPs and MLNs, the spleen IgM^+^ B cells were isolated and stimulated by IL-21. The result demonstrated that IL-21 did promote IgA production and IgA CSR in spleen IgM^+^ B cells (Supplementary Figure 3).

### IL-21 Facilitates Porcine Plasma Cell Differentiation

**Figure 4:**
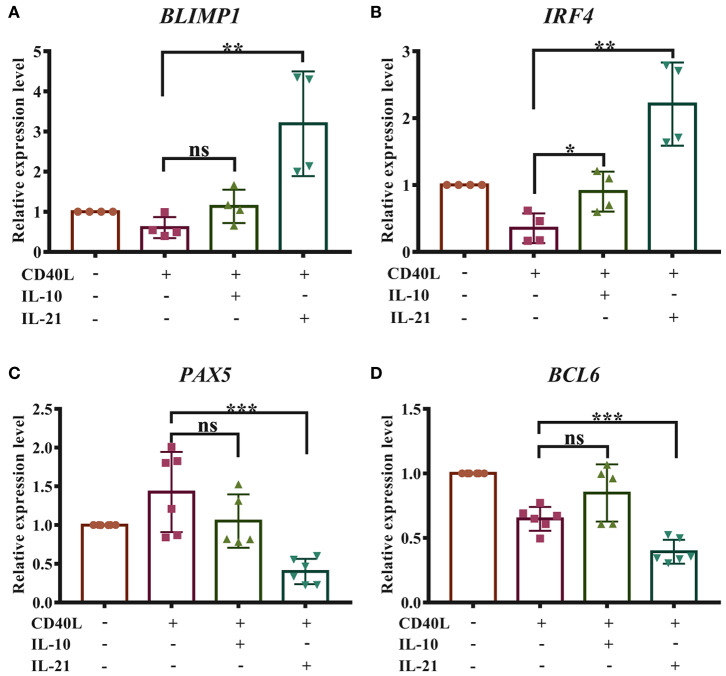
IL-21 facilitates the differentiation of porcine plasma cells. Total RNA was extracted from IgM^+^ B cells after 72 h of culture with CD40L/IL-10/IL-21. Real-time quantitative PCR was performed to analyze the relative expression levels of *BLIMP1* **(A)**, *IRF4* **(B)**, *PAX5* **(C)**, and *BCL6* **(D)**, normalized by *GAPDH*. Cumulative data from three independent experiments were analyzed and presented. **p ≤ 0.01; ***p ≤ 0.001.

The differentiation of plasma cells was regulated by several transcription factors. B-lymphocyte-induced maturation protein 1 (BLIMP1) and interferon regulatory factor 4 (IRF4) have been considered as important regulators for PC differentiation, while Bcl6 and Pax5 actively repress the BLIMP1 expression and favor the generation of proliferating B cells in the germinal center (16). To investigate whether IL-10 and IL-21 influence the differentiation of plasma cells, the relative expression levels of *BLIMP1, IRF4, BCL6*, and *PAX5* were measured by real-time RT-qPCR, and the results revealed that IL-21 significantly increased the expression of *BLIMP1* (Figure 4A) and *IRF4* (Figure 4B) and decreased the expression of *PAX5* (Figure 4C) and *BCL6* (Figure 4D). However, IL-10 showed less impact on the expression of these regulators. These results suggested that IL-21 contributed to plasma cell differentiation, resulting in increased antibody production.

### IL-21 Activates the JAK-STAT Signaling Pathway

**Figure 5:**
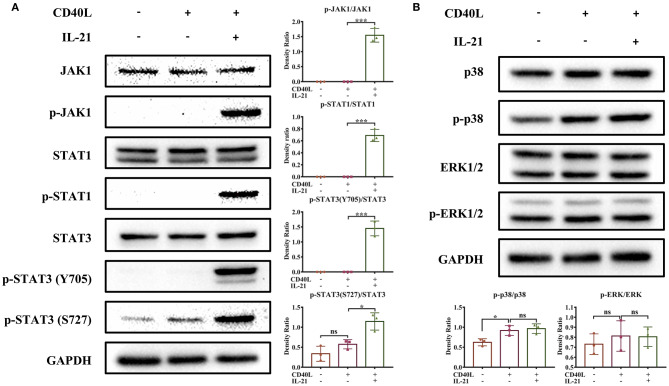
IL-21 activates JAK1, STAT1, and STAT3 in IgM^+^ B cells. IgM^+^ B cells from Peyer's patches were cultured with or without CD40L overnight and then stimulated with or without IL-21 for 2 h. Western blotting was performed to detect total or phosphorylated proteins involved in JAK-STAT, MAPK, and PI3K-AKT signaling pathways. Representative plots of three independent experiments for the detection of total or phosphorylated JAK1, STAT1, and STAT3 proteins **(A)**, as well as p38 and ERK1/2 proteins **(B)**, are shown. The cumulative density ratio of phosphorylated protein normalized by total protein is also shown. *p ≤ 0.05; ***p ≤ 0.001.

To identify the signaling pathways and associated molecules activated by IL-21 in porcine B cells, total and phosphorylated levels of key proteins in Janus kinase and signal transducer and activator of transcription (JAK-STAT), phosphoinositide 3-kinase (PI3K), and mitogen-activated protein kinase (MAPK) pathways were evaluated by western blotting. The results showed that IL-21 significantly upregulated the phosphorylation levels of JAK1, STAT1, and STAT3 proteins in porcine B cells (Figure 5A). However, the JAK2, JAK3, Tyk2, STAT5, and STAT6 proteins were not phosphorylated in the same samples (data not shown). IL-21 did not increase the phosphorylation levels of MAPK p38 and ERK1/2 proteins in MAPK pathways (Figure 5B), as well as AKT or PI3K p85 proteins in PI3K signaling pathways (data not shown) in porcine B cells. All these results indicated that IL-21R signaling activated JAK-STAT pathways, predominantly JAK1, STAT1, and STAT3, in porcine IgM^+^ B cells.

### The JAK-STAT Signaling Pathway Is Involved in IgA CSR of Porcine B Cells

**Figure 6:**
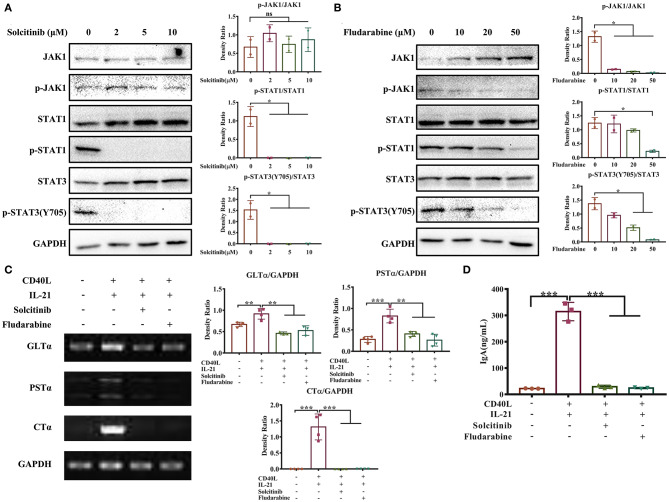
The JAK-STAT signaling pathway is involved in IL-21-induced IgA CSR. IgM^+^ B cells from porcine Peyer's patches were cultured with different concentrations of solcitinib **(A)** or fludarabine **(B)** for 24 h and then stimulated with CD40L+IL21 for 2 h. The total and phosphorylated amounts of JAK1, STAT1, and STAT3 proteins were assessed by western blotting, and their density ratio was also analyzed and shown. IgM^+^ B cells were treated with 2 μM solcitinib or 50 μM fludarabine for 24 h and were then cultured with CD40L+IL-21 for 5 days. The cells were then collected at 60 h post-treatment and subjected to semi-quantitative PCR to assess the expression levels of GLTα, PSTα, and CTα **(C)**, while cell supernatants were collected, and IgA levels were detected by ELISA **(D)**. All experiments were performed independently in duplicate or triplicate. *p ≤ 0.05; **p ≤ 0.01; ***p ≤ 0.001.

Next, we investigated whether the JAK-STAT signaling pathway activated by IL-21 was involved in IgA CSR of porcine B cells. Two inhibitors targeting the JAK-STAT signaling pathway were employed; the concentration of each inhibitor was optimized, and the results showed that 2 μM solcitinib inhibited the phosphorylation of STAT1 and STAT3 proteins (Figure 6A), while 50 μM fludarabine inhibited the phosphorylation of JAK1, STAT1, and STAT3 proteins (Figure 6B). However, the MAPK signaling pathways were not inhibited by either inhibitor at any concentration (Supplementary Figure 4). Upon treating cells with the optimal concentrations of inhibitors (2 μM solcitinib or 50 μM fludarabine) and then stimulating with IL-21, the IgA CSR in porcine B cells and IgA production were evaluated. The results demonstrated that the expression levels of GLTα, PSTα, and CTα were upregulated by IL-21 stimulation but inhibited by either solcitinib or fludarabine (Figure 6C). Similarly, the IgA secretion from IgM^+^ B cells was significantly increased by IL-21 induction but dramatically decreased to basal levels when treating with either inhibitor (Figure 6D). Collectively, these data suggested that the JAK-STAT signaling pathway was involved in IgA CSR and IgA production of porcine B cells.

## Discussion

Peyer's patches are considered the most important IgA-producing gut-associated lymphoid tissues (17). In this study, we found that IL-21 played an important role in T cell-dependent proliferation and differentiation of IgA-producing cells in porcine Peyer's patches, mainly through the JAK-STAT signaling pathway. To our knowledge, this is the first report illuminating the efficacy and mechanisms of cytokines on the differentiation of IgA^+^ B cells in porcine Peyer's patches. In previous studies, the IgM and IgA transcripts, which contain VDJ and C regions, were cloned and hybridized with specific probes to measure repertoire diversification and CSR in pigs (18, 19). In this study, three direct indicators, GLTα, PSTα, and CTα, were detected by (nest-)PCR methods for the first time to evaluate IgA CSR in porcine B cells, which is simpler and less time-consuming to accomplish.

BAFF and APRIL, mainly produced by DCs, have been shown to induce T cell-independent IgA CSR in the gut (20, 21). Our results demonstrated that BAFF and APRIL also exhibited favorable effects on IgA production in porcine B cells of Peyer's patches, showing their conservative regulatory roles in IgA production among species. CD40L, which binds to CD40 expressed on B cells, is considered to provide a TD signal. Interleukins and TGF-β1, mainly produced by T cells, combine with CD40L to stimulate TD IgA generation. TGF-β1 induces potent IgA CSR in mouse models, and it strongly promotes IL-21-mediated IgA CSR in humans (22, 23). In our results, TGF-β1 alone showed a limited impact on porcine IgA production and impaired porcine B cell proliferation (data not shown), which is recognized to be a prerequisite for IgA CSR (24), whether it plays an indirect role in IgA production requires further investigation. IL-6 induces IgA secretion in mice (25, 26) but failed to increase IgA production in pigs, indicating that IL-6 may act as a cofactor in IgA production or function differently in IgA CSR between mouse and pig. IL-10 significantly increased porcine B cell proliferation and IgA production but showed finite effects on IgA CSR and plasma cell differentiation. The detailed mechanism responsible for this phenotype needs further investigation.

IL-21, a type 1 cytokine mainly produced by CD4^+^ T cells and natural killer T cells (NKT), shows pleiotropic effects on a variety of immune cells. IL-21 binds to the receptor IL-21R dominantly expressed on B cells and enhances B cell proliferation, maturation, plasma cell and memory B cell differentiation, and immunoglobulin class switching in mouse models and human studies (27–29). A previous study showed that IL-21 drove the proliferation and differentiation of porcine spleen B cells into antibody-secreting cells (30). In this study, we found that exogenous IL-21 combined with CD40L significantly upregulated the IgA production level in porcine B cells through the promotion of proliferation, PC differentiation and IgA CSR of B cells, which was consistent with the findings in humans (31, 32). IL-21 itself does not facilitate IgA CSR in mouse; instead, it augments TGF-β- or RA-mediated IgA CSR, indicating that mouse IL-21 may act as a cofactor in promoting IgA production in mouse (33). These findings illuminated that IL-21 plays a conservative role in IgA generation between pigs and humans, but that role differs in the mouse.

As reported previously, IL-21 activates multiple signaling pathways, including JAK-STAT, PI3K and MAPK pathways (34). In consistent with the findings from humans, we discovered that IL-21 predominantly promoted the phosphorylation of JAK1, STAT1, and STAT3 proteins in the JAK-STAT pathway in porcine B cells. Moreover, our results further indicated that the JAK-STAT signaling pathway was involved in IL-21-mediated IgA CSR. However, the lack of specific inhibitors of JAK1/STAT1/STAT3 impedes investigations about the detailed mechanisms responsible for the JAK-STAT signaling pathway in swine mucosal immunity.

Previous studies have demonstrated that the percentage of CD21^+^ B cells in porcine IPP decreases from higher than 90 to 40–60%, while the percentage of CD4^+^ Th cell increases from 1–3 to 6–10% as pigs grow from 1 to 3–4 months old, indicating that more Th cells develop or migrate into porcine IPP with the increase in age (5, 6, 12, 13). In addition, Andersen et al. revealed that CD40L (CD154), mainly expressed on T cells, rescues porcine IPP follicular B cells from apoptosis and facilitates their maturation (12, 13). Our findings in this study also showed that B cells only accounted for 20–40% of the IPPs of adult pigs and were sensitive to apoptosis. CD40L promoted the proliferation of B cells, increasing the live cell percentage. With the help of IL-21, live B cells undergo further proliferation and differentiation to IgA-producing plasma cells, indicating that T cell-dependent IgA CSR may occur in porcine IPP with the action of cytokines such as IL-21. Taken together, we speculate that IPPs are T-cell dependent, IgA-producing sites in young and adult pigs.

Naive and memory B cells are reported to have different mechanisms of differentiation to antibody-secreting plasma cells (35). However, in this study, a universal surface marker expressed on both naive and memory B cells, IgM, was used for B cell isolation. To better understand the mucosal immune response of naive porcine B cells, more specific markers are required to sort naive B cells in high purity.

Orally delivered vaccines induce higher intestinal IgA levels than conventional vaccination strategies, representing a promising method to prevent enteric pathogens. Due to the unique features of the mucosal membrane, effective adjuvant and appropriate delivery vectors are highly demanded to improve the efficacy of the mucosal vaccine (36). This study provided vital information that adjuvant or reagent that increases the IL-21 expression in porcine Peyer's patches may improve the intestinal mucosal immunity of vaccines and IgA production in the gut lumen.

**Figure 7:**
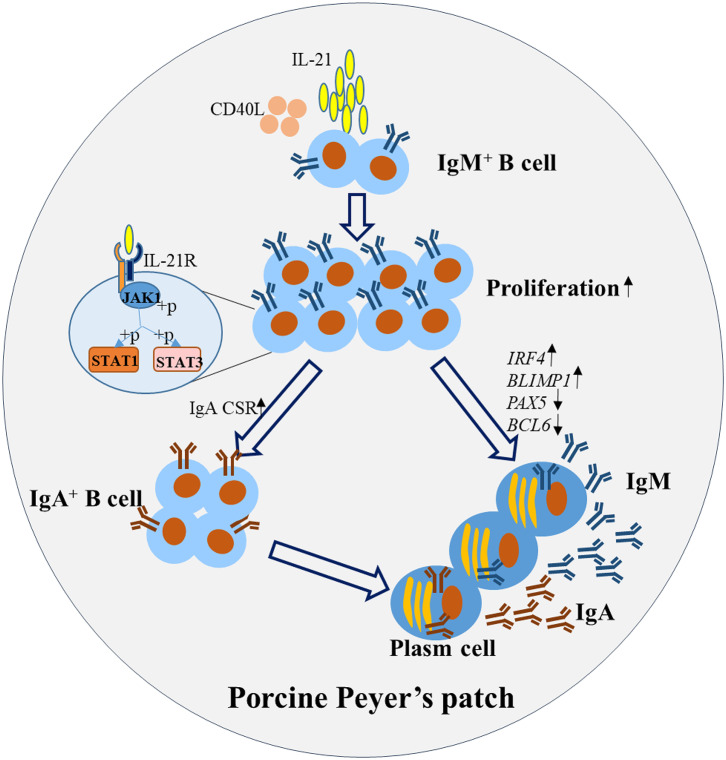
Proposed working model for IL-21-induced B cell differentiation and IgA-producing plasma cells in porcine Peyer's patches. IL-21 binds to the IL-21R on B cells, promotes the proliferation and inhibits the apoptosis. IL-21 increased IgA CSR, through the phosphorylation of JAK1, STAT1, and STAT3 leading to the production of IgA^+^ B cells. Additionally, IL-21 promoted the differentiation of plasma cells, characterized by increased expression of *BLIMP1* and *IRF4* and decreased expression of *PAX5* and *BCL6*. Thus, IgA was robustly produced by the IgA-secreting B cells in porcine Peyer's patches.

In summary, this study illustrated the mechanism for IL-21-induced TD IgA CSR and antibody production in porcine ileum Peyer's patches. The detailed pathways are summarized and presented in Figure 7. Briefly, IL-21 binds to the IL-21R on B cells, activates their proliferation and induces the phosphorylation of JAK1, STAT1, and STAT3. IL-21 promotes plasma cell differentiation and increases IgA CSR in IgM^+^ B cells through the JAK-STAT signaling pathway, leading to the production of IgA-secreting B cells. Our discoveries from this study will contribute to porcine mucosal immunology and provide novel insights to increase IL-21 expression levels in porcine Peyer's patches to boost IgA production for the prevention of enteric pathogens in mucosal vaccine design.

## Data Availability Statement

The datasets generated for this study can be found in the NCBI GenBank under the accession numbers MN629331 (GLTα), MN629332 and MN629333 (PSTα), MN629334 (CTα).

## Ethics Statement

The animal study was reviewed and approved by Laboratory Animals of Lanzhou Veterinary Research Institute, Chinese Academy of Agricultural Sciences, China.

## Author Contributions

GuaL and GuoL designed the experiments, analyzed the data, and wrote and edited the manuscript. GuoL performed the experiments. BW, QC, YL, BL, NY, SY, and SG did animal work and collected the samples. All authors contributed to the article and approved the submitted version.

## Conflict of Interest

The authors declare that the research was conducted in the absence of any commercial or financial relationships that could be construed as a potential conflict of interest.

## References

[1] Mantis NJ Rol N Corthesy B. Secretory IgA's complex roles in immunity and mucosal homeostasis in the gut. Mucosal Immunol. (2011) 4:603–11. 10.1038/mi.2011.41 21975936 PMC3774538

[2] Lycke NY Bemark M. The regulation of gut mucosal IgA B-cell responses: recent developments. Mucosal Immunol. (2017) 10:1361–74. 10.1038/mi.2017.62 28745325

[3] Butler JE Sinkora M. The enigma of the lower gut-associated lymphoid tissue (GALT). J Leukoc Biol. (2013) 94:259–70. 10.1189/jlb.0313120 23695307

[4] Butler JE Santiago-Mateo K Wertz N Sun X Sinkora M Francis DL. Antibody repertoire development in fetal and neonatal piglets. XXIV hypothesis: the ileal Peyer patches (IPP) are the major source of primary, undiversified IgA antibodies in newborn piglets. Dev Comp Immunol. (2016) 65:340–51. 10.1016/j.dci.2016.07.020 27497872

[5] Obremski K Podlasz P Zmigrodzka M Winnicka A Wozny M Brzuzan P. The effect of T-2 toxin on percentages of CD4+, CD8+, CD4+ CD8+ and CD21+ lymphocytes, and mRNA expression levels of selected cytokines in porcine ileal Peyer's patches. Pol J Vet Sci. (2013) 16:341–9. 10.2478/pjvs-2013-0046 23971203

[6] Obremski K. The effect of *in vivo* exposure to zearalenone on cytokine secretion by Th1 and Th2 lymphocytes in porcine Peyer's patches after *in vitro* stimulation with LPS. Pol J Vet Sci. (2014) 17:625–32. 10.2478/pjvs-2014-0093 25638976

[7] Yuan C Zhang E Huang L Wang J Yang Q. Oral administration of inactivated porcine epidemic diarrhea virus activate DCs in porcine Peyer's patches. BMC Vet Res. (2018) 14:239. 10.1186/s12917-018-1631-9 30115049 PMC6097195

[8] Jing Y Liu H Xu W Yang Q. 4,4'-Diaponeurosporene-producing bacillus subtilis promotes the development of the mucosal immune system of the piglet gut. Anat Rec. (2019) 302:1800–7. 10.1002/ar.24102 30809953

[9] Stavnezer J Schrader CE. IgH chain class switch recombination: mechanism and regulation. J Immunol. (2014) 193:5370–8. 10.4049/jimmunol.1401849 25411432 PMC4447316

[10] Estes DM. Regulation of IgA responses in cattle, humans and mice. Vet Immunol Immunopathol. (2010) 138:312–7. 10.1016/j.vetimm.2010.10.009 21074276

[11] Butler JE Wertz N Sinkora M. Antibody repertoire development in swine. Annu Rev Anim Biosci. (2017) 5:255–79. 10.1146/annurev-animal-022516-022818 28199170

[12] Andersen JK Takamatsu H Oura CA Brookes SM Pullen L Parkhouse RE. Systematic characterization of porcine ileal Peyer's patch, I. Apoptosis-sensitive immature B cells are the predominant cell type. Immunology. (1999) 98:612–21. 10.1046/j.1365-2567.1999.00922.x 10594696 PMC2326965

[13] Andersen JK Takamatsu H Pullen L Parkhouse RM. Systematic characterization of porcine ileal Peyer's patch, II. A role for CD154 on T cells in the positive selection of immature porcine ileal Peyer's patch B cells. Immunology. (1999) 98:622–9. 10.1046/j.1365-2567.1999.00923.x 10594697 PMC2326978

[14] Soldevila F Edwards JC Graham SP Stevens LM Crudgington B Crooke HR. Characterization of the myeloid cell populations' resident in the porcine palatine tonsil. Front Immunol. (2018) 9:1800. 10.3389/fimmu.2018.01800 30158925 PMC6104124

[15] Duvigneau JC Hartl RT Groiss S Gemeiner M. Quantitative simultaneous multiplex real-time PCR for the detection of porcine cytokines. J Immunol Methods. (2005) 306:16–27. 10.1016/j.jim.2005.06.021 16223507

[16] Owen JA Punt J Stranford SA Jones PP Kuby J Kuby Immunology. New York, NY: W. H. Freeman and Company (2013).

[17] Cerutti A Rescigno M. The biology of intestinal immunoglobulin A responses. Immunity. (2008) 28:740–50. 10.1016/j.immuni.2008.05.001 18549797 PMC3057455

[18] Butler JE Santiago-Mateo K Sun XZ Wertz N Sinkora M Francis DH. Antibody repertoire development in fetal and neonatal piglets. XX B cell lymphogenesis is absent in the ileal Peyer's patches, their repertoire development is antigen dependent, and they are not required for B cell maintenance. J Immunol. (2011) 187:5141–9. 10.4049/jimmunol.1101871 22013126

[19] Butler JE Sun X Wertz N Vincent AL Zanella EL Lager KM. Antibody repertoire development in fetal and neonatal piglets. XVI Influenza stimulates adaptive immunity, class switch and diversification of the IgG repertoire encoded by downstream Cgamma genes. Immunology. (2013) 138:134–44. 10.1111/imm.12018 23320646 PMC3575766

[20] Tezuka H Abe Y Iwata M Takeuchi H Ishikawa H Matsushita M. Regulation of IgA production by naturally occurring TNF/iNOS-producing dendritic cells. Nature. (2007) 448:929–33. 10.1038/nature06033 17713535

[21] Massacand JC Kaiser P Ernst B Tardivel A Burki K Schneider P. Intestinal bacteria condition dendritic cells to promote IgA production. PLoS ONE. (2008) 3:e2588. 10.1371/journal.pone.0002588 18596964 PMC2432026

[22] Dullaers M Li D Xue Y Ni L Gayet I Morita R. A T cell-dependent mechanism for the induction of human mucosal homing immunoglobulin A-secreting plasmablasts. Immunity. (2009) 30:120–9. 10.1016/j.immuni.2008.11.008 19144318 PMC2659635

[23] Stavnezer J Kang J. The surprising discovery that TGF beta specifically induces the IgA class switch. J Immunol. (2009) 182:5–7. 10.4049/jimmunol.182.1.5 19109126

[24] Tangye SG Hodgkin PD. Divide and conquer: the importance of cell division in regulating B-cell responses. Immunology. (2004) 112:509–20. 10.1111/j.1365-2567.2004.01950.x 15270721 PMC1782517

[25] Ramsay AJ Husband AJ Ramshaw IA Bao S Matthaei KI Koehler G. The role of interleukin-6 in mucosal IgA antibody responses *in vivo*. Science. (1994) 264:561–3. 10.1126/science.8160012 8160012

[26] Sato A Hashiguchi M Toda E Iwasaki A Hachimura S Kaminogawa S. CD11b+ Peyer's patch dendritic cells secrete IL-6 and induce IgA secretion from naive B cells. J Immunol. (2003) 171:3684–90. 10.4049/jimmunol.171.7.3684 14500666

[27] Ding BB Bi E Chen H Yu JJ Ye BH. IL-21 and CD40L synergistically promote plasma cell differentiation through upregulation of Blimp-1 in human B cells. J Immunol. (2013) 190:1827–36. 10.4049/jimmunol.1201678 23325890 PMC3563840

[28] Davis MR Zhu Z Hansen DM Bai Q Fang Y. The role of IL-21 in immunity and cancer. Cancer Lett. (2015) 358:107–14. 10.1016/j.canlet.2014.12.047 25575696

[29] Robinson MJ Pitt C Brodie EJ Valk AM O'Donnell K Nitschke L. BAFF, IL-4 and IL-21 separably program germinal center-like phenotype acquisition, BCL6 expression, proliferation and survival of CD40L-activated B cells *in vitro*. Immunol Cell Biol. (2019) 97:826–39. 10.1111/imcb.12283 31276232

[30] Rahe MC Murtaugh MP. Interleukin-21 drives proliferation and differentiation of porcine memory B cells into antibody secreting cells. PLoS ONE. (2017) 12:e0171171. 10.1371/journal.pone.0171171 28125737 PMC5268775

[31] Borte S Pan-Hammarstrom Q Liu C Sack U Borte M Wagner U. Interleukin-21 restores immunoglobulin production *ex vivo* in patients with common variable immunodeficiency and selective IgA deficiency. Blood. (2009) 114:4089–98. 10.1182/blood-2009-02-207423 19738033

[32] Avery DT Deenick EK Ma CS Suryani S Simpson N Chew GY. B cell-intrinsic signaling through IL-21 receptor and STAT3 is required for establishing long-lived antibody responses in humans. J Exp Med. (2010) 207:155–71. 10.1084/jem.20091706 20048285 PMC2812540

[33] Cao AT Yao S Gong B Nurieva RI Elson CO Cong Y. Interleukin (IL)-21 promotes intestinal IgA response to microbiota. Mucosal Immunol. (2015) 8:1072–82. 10.1038/mi.2014.134 25586558 PMC4501922

[34] Leonard WJ Wan CK. IL-21 signaling in immunity. F1000Res. (2016) 5:F1000 Faculty Rev-224. 10.12688/f1000research.7634.1 26966515 PMC4770986

[35] Deenick EK Avery DT Chan A Berglund LJ Ives ML Moens L. Naive and memory human B cells have distinct requirements for STAT3 activation to differentiate into antibody-secreting plasma cells. J Exp Med. (2013) 210:2739–53. 10.1084/jem.20130323 24218138 PMC3832925

[36] Boyaka PN. Inducing mucosal IgA: a challenge for vaccine adjuvants and delivery systems. J Immunol. (2017) 199:9–16. 10.4049/jimmunol.1601775 28630108 PMC5719502

