# Supplementary material for: Interleukin (IL)-21 Promotes the Differentiation of IgA-Producing Plasma Cells in Porcine Peyer's Patches via the JAK-STAT Signaling Pathway
Source: Front Immunol. 2020 Jun 23;11:1303. doi: 10.3389/fimmu.2020.01303 (PMC7324671; doi:10.3389/fimmu.2020.01303)

Supplementary materials

Supplementary Figure S1. Preparation and identification of IgM$^+$ B cells from porcine Peyer's patches. Total lymphocytes were isolated from porcine Peyer's patches and incubated with IgM primary antibody for 30min at 4℃, followed by Alexa Fluor® 647 conjugated anti-mouse IgG (Abcam). IgM$^+$ B cells magnetically were separated. 7-AAD (BD Bioscience) was used to label nonviable cells. The percentage of IgM$^+$ B cells in total lymphocytes or purified IgM$^+$ cells were tested by flow cytometry. (C-D) Representative flow cytometry plots showing the percentage of IgM$^+$ B cells in total PP lymphocytes (C) and in purified PP IgM$^+$ cells (D).

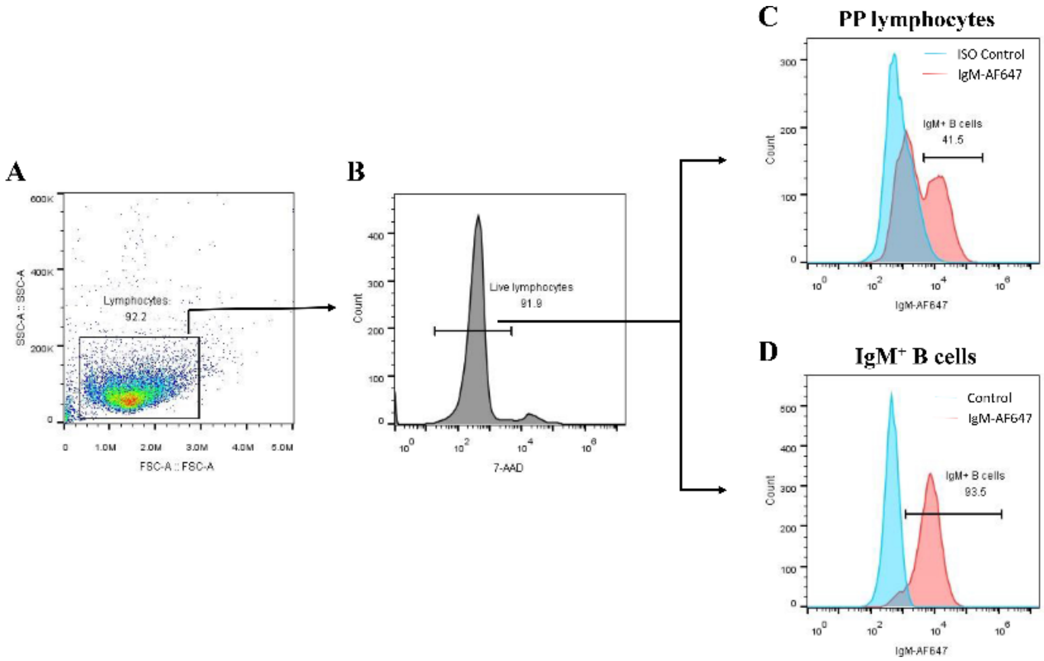

Supplementary Figure S2. Sequence analysis of porcine GLTα, PSTα and CTα. Porcine GLTα, PSTα and CTα were amplified by PCR and linked to sequencing vector, followed by sequencing. The sequences were aligned with Sus scrofa IgH constant region gene sequences. The sequence structures of these three molecular markers for porcine IgA CSR were shown.

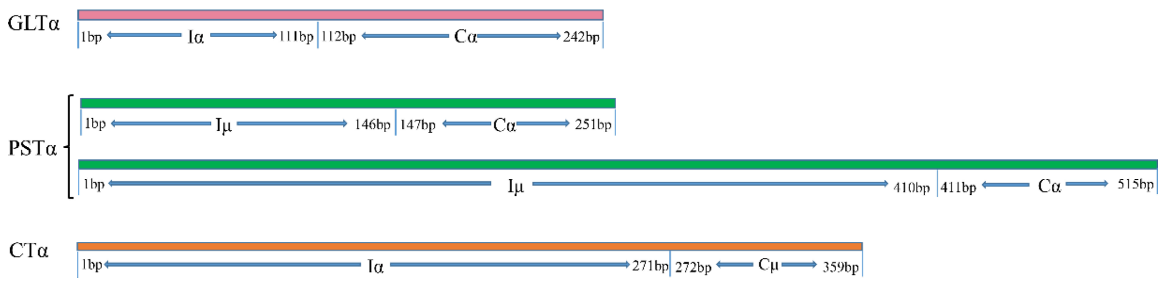

Supplementary Figure S3. IL-21 promotes IgA production and IgA class switch recombination in porcine spleen B cells. IgM^{+} B cells were isolated from porcine spleen and cultured with or without CD40L and IL-21. IgA levels in the supernatant were measured at 6 days post stimulation by ELISA (A) and all the negative values are zeroed during analyzing. The expression level of GLTα，PSTα, and CTα were detected at 3 days post treatment (B) and all these PCR products were verified by DNA sequencing. Additionally, their density ratio were normalized by *GAPDH* and calculated (C).

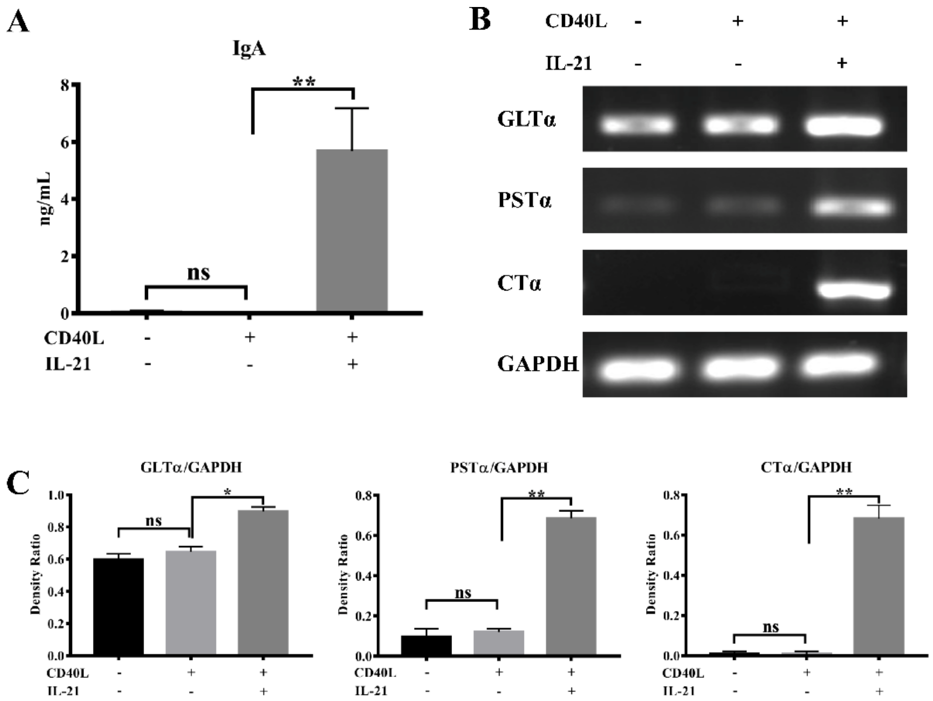

Supplementary Figure S4. MAPK signaling pathway is not inhibited by Solcitinib and Fludarabine. IgM$^+$ B cells were treated with different concentrations of Solcitinib (A) or Fludarabine (B) for 24 hours, then stimulated with CD40L+IL21 for 2 hours. The total amount as well as phosphorylated P38 and ERK1/2 proteins were detected by western blot.

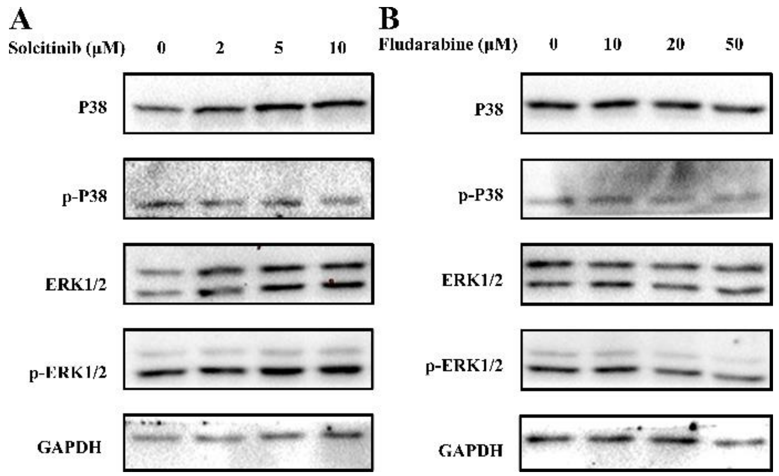

Supplement: Supplementary file 1 [file Image_1.pdf]
